# Development of a Tumor‐Bearing Animal Model to Evaluate Chemotherapy Efficacy and Toxicity

**DOI:** 10.1002/cnr2.70618

**Published:** 2026-07-01

**Authors:** Ifeoma J. Dikeocha, Emma Bateman, Hannah R. Wardill, Joanne M. Bowen

**Affiliations:** ^1^ Discipline of Physiology, School of Pharmacy and Biomedical Sciences Adelaide University Adelaide South Australia Australia; ^2^ Supportive Oncology Research Group, Precision Cancer Medicine The South Australian Health and Medical Research Institute Adelaide Australia

## Abstract

**Background:**

The value of cancer therapies lies in enhancing anti‐tumor efficacy while minimizing toxicity. However, these aspects are rarely studied together, often due to the challenges of modelling both within the same framework. In preclinical settings, rat models like the Dark Agouti Mammary Adenocarcinoma (DAMA) model are used to evaluate both efficacy and toxicity concurrently but are limited in duration, relying on a single dose to evaluate efficacy and toxicity outcomes.

**Aim:**

This study aimed to develop a cyclical chemotherapy model using the DAMA model to better mimic multi‐cycle clinical scenarios.

**Methods:**

Methotrexate (MTX) was administered in various dosing schedules to assess tumor control and animal welfare.

**Results:**

A dose of 2 mg/kg administered once weekly provided sufficient tumor control and the longest survival (14.25 ± 2.87 days), while maintaining animal welfare and yielding an acceptable efficacy‐to‐toxicity ratio. Dosing frequency, rather than cumulative dose, had a greater impact on welfare, as 2 mg/kg MTX once weekly substantially affected animal welfare, whereas the total cumulative dose of 4 mg/kg MTX did not.

**Conclusion:**

These findings will be utilized in future studies testing interventions designed for preventing chemotherapy toxicity whilst maintaining tumor efficacy.

## 1 Introduction

The value of cancer treatment lies in its ability to demonstrate tumor efficacy with acceptable toxicity, with both measures critical to optimize outcomes for people with cancer [1]. For instance, any supportive care intervention must be evaluated for its potential to impair the efficacy of chemotherapy, and new anti‐tumor drugs should be thoroughly tested for their toxicity potential. However, it is rare for these factors to be investigated together, with separate models focusing on each outcome in isolation, reflecting the inherent challenges in modelling both ends of the treatment spectrum within the same framework.

Rodent models of cancer and its treatment are commonly employed to develop and test new therapies, with a plethora of models available each with their own set of unique advantages and challenges [2]. The Dark Agouti Mammary Adenocarcinoma (DAMA) model is a long‐standing, tumor‐bearing rodent model in which both efficacy and toxicity can be concurrently evaluated [3]. Its historical use has almost exclusively focused on mucositis, whilst also ensuring nutritional interventions do not interfere with intended anti‐cancer effects [4]. Whilst its utility in this context is undisputed, it is undeniably limited by its short duration and reliance on a single cycle of chemotherapy, which fails to recapitulate the cyclical nature of most cancer therapy regimens.

In the context of chemotherapy, modeling both efficacy and toxicity in a clinically‐relevant manner requires agents to be administered in cycles, with each cycle contributing to the cumulative dose required for the optimal depth and duration of tumor control [5]. However, replicating this cyclical administration in animal cancer studies presents significant challenges related to animal welfare, resistance and variable tumor dynamics [6]. As such, we aimed to identify a cyclical chemotherapy dosing schedule in the DAMA model that allows for sustained tumor control, with mild‐to‐moderate toxicity profiles, allowing both efficacy and toxicity to be evaluated concurrently and in a more clinically relevant manner.

## 2 Materials and Methods

This study was approved by the Animal Ethics Committee at the University of Adelaide (M‐2023‐018) in accordance with National Health and Research Council (Australia) NHMRC guidelines for use of laboratory animals.

Female DA rats (*n* = 24) weighing 140–160 g were sourced from Ozgene Australia. Rats were pair housed in ventilated cages under a 12 h light/dark cycle and a constant temperature of 20°C–23°C, with *ad libitum* access to water and AIN93G diet. 2 × 10^6^ DAMA cells were implanted subcutaneously into the left and right flank of passage rats as previously described [7]. Tumors in the exponential growth phase were harvested; DAMA cells were isolated and implanted into all experimental rats. Once palpable, tumors were measured daily using digital calipers to determine their volume ([length× width× depth] × [π/6] expressed as cm^3^). Tumor burden was calculated as tumor volume relative to body weight (%BW, cm^3^/g). The chemotherapeutic agent, methotrexate (MTX), was used in this model as DAMA is reliably sensitive to it [8]. MTX stock concentration of 25 mg/mL diluted in saline was administered intramuscularly (i.m.) when tumors were between 0.3% and 0.5%BW. Welfare and body weight were assessed daily, with diarrhea graded using a well‐established system previously reported [9]. Rats were euthanized if tumors reached 10%BW (efficacy endpoint) or weight loss > 15% (toxicity endpoint).

MTX was administered at different doses and frequencies (Table 1), each aiming to control tumor growth (efficacy) whilst maintaining animal welfare (toxicity). Dosing duration was not predefined but instead dictated based on animal welfare, with dosing ongoing until a humane endpoint was reached. Hence the total/cumulative dose of MTX received by each rat was variable within rat groups. This study was done in 3 cohorts. In each cohort, tumor burden at cull, weight at cull, and days of survival was analyzed by Unpaired *t*‐test, GraphPad prism v10.

**TABLE 1:** MTX dosing schedules.

	Schedule	Methotrexate dose (*n*)	Dose frequency
Cohort 1	1	2 mg/kg (*n* = 4)	Every 4 days
	2	1.5 mg/kg (*n* = 4)	Every 4 days
Cohort 2	3	0.5 mg/kg (*n* = 4)	Every 3 days
	4	0.75 mg/kg (*n* = 4)	Every 3 days
Cohort 3	5	2.5 mg/kg (*n* = 4)	Every 7 days
	6	2 mg/kg (*n* = 4)	Every 7 days

## 3 Results

**FIGURE 1:**
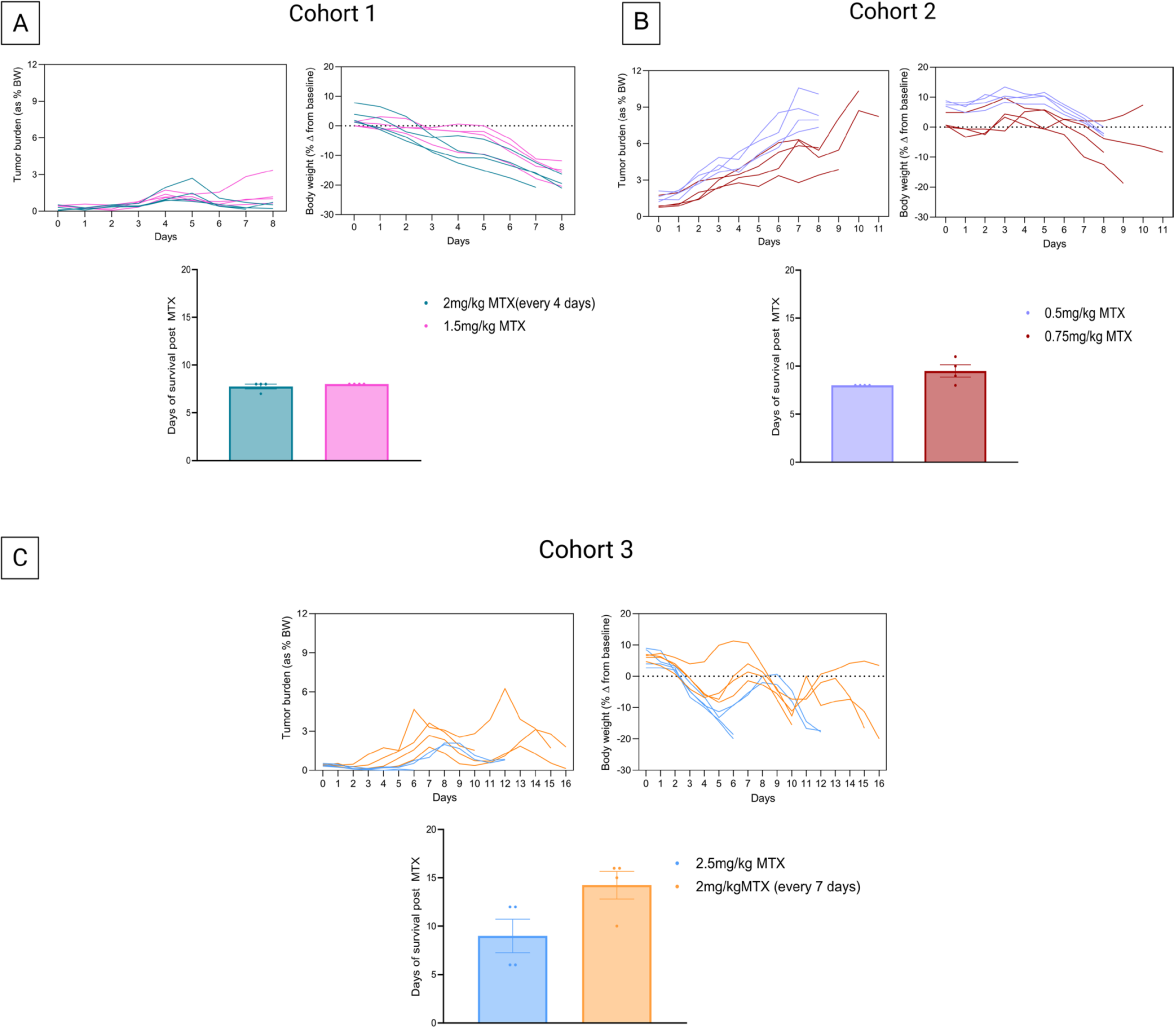
A: Cohort 1, B: Cohort 2, C: Cohort 3; Showing Tumor burden over time, %Change of bodyweight over time, and Days of survival per group. Data shown as mean ± SEM (Day 0 = First MTX).

**FIGURE 2:**
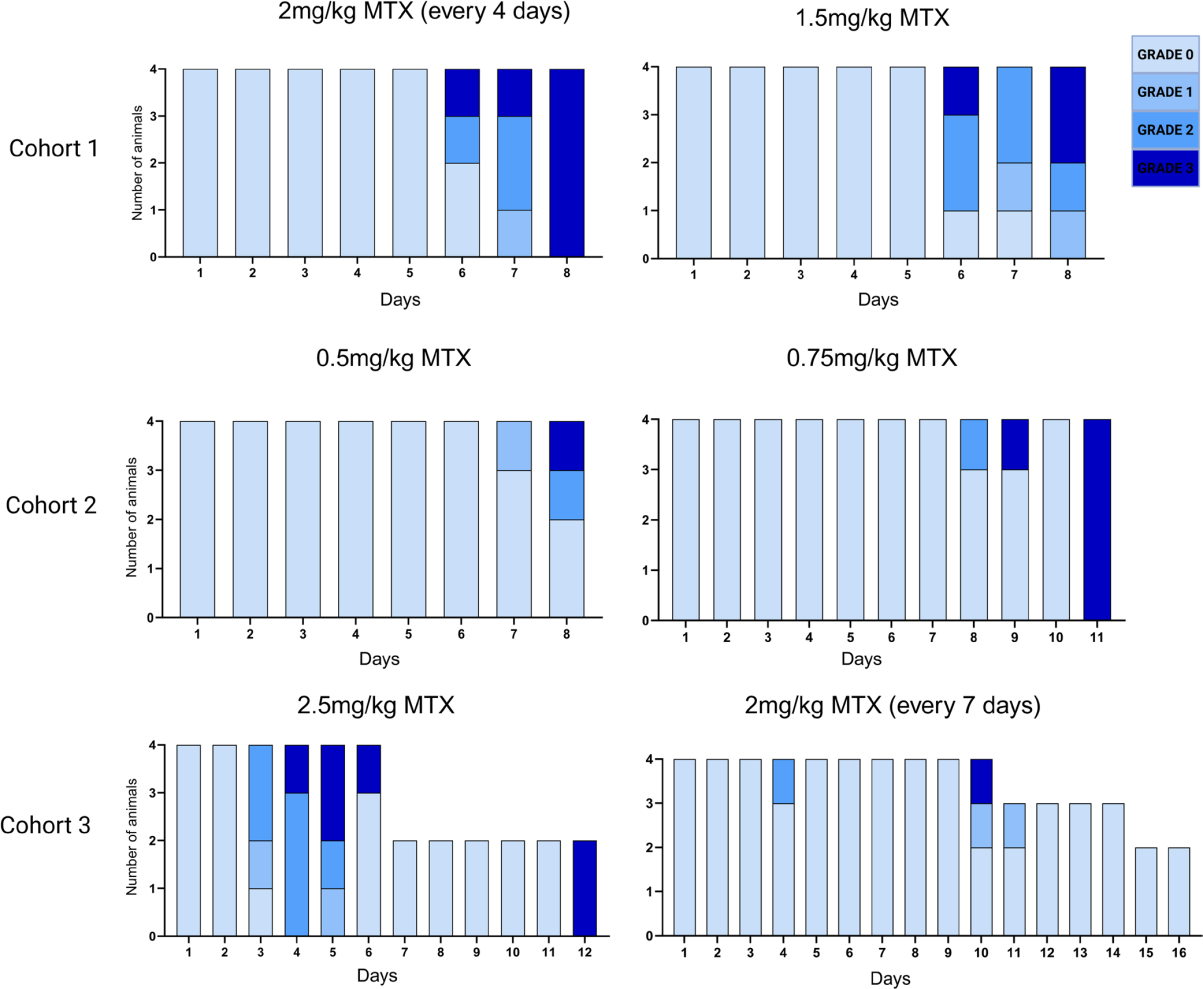
Diarrhea proportions per group. Data expressed as observation of diarrhea in each dose schedule (Day 0 = First MTX).

In the first cohort, Dosing schedule 1 (2 mg/kg, every 4 days) resulted in high tumor control (Figure 1A) at the expense of animal welfare indicated by weight loss and grade 3 diarrhea (Figure 2). As a result, all rats were euthanized after 2 doses of MTX. Schedule 2 (1.5 mg/kg, every 4 days). Even at a reduced dose, tumor control was observed (Figure 1A) but welfare remained unacceptable, with all rats euthanized due to weight loss on day 8.

The cumulative dose of MTX administered in Schedules 1 and 2 was high, and whilst there was tumor control, welfare was unacceptable. We tried to overcome this in cohort 2 where we adopted considerably reduced MTX doses (0.5 mg/kg and 0.75 mg/kg), opting for a higher frequency to address rapid tumor growth. In the 0.5 mg/kg group, while welfare implications were improved, tumor control was lessened (Figure 1B), with tumors growing with little to no plateau in size after MTX administration, and as a result rats were euthanized on day 8. This showed relatively minimal toxicity implications in schedule 3, and a modestly higher MTX dose (0.75 mg/kg) in Schedule 4 resulted in tumors reaching 10%BW with delayed weight loss, and an average survival rate of 9.5 ± 1.29 days.

Considering the slightly improved survival and welfare observed in Schedule 4, we opted for a higher MTX dose but administered less frequently, investigating 2.5 mg/kg and 2 mg/kg in cohort 3. Schedule 5 (2.5 mg/kg, every 7 days) controlled tumors but also induced rapid weight loss (Figure 1C), leading to a mean survival of 9.0 ± 3.46 days. While schedule 6 (2 mg/kg, every 7 days) showed tumor control, with one rat recovering to their baseline weight and achieving the longest survival of all schedules 14.25 ± 2.87 days (Figure 1C).

No significant difference was found between the groups in all three cohorts when tumor burden at cull, weight at cull, and overall days of survival were statistically analyzed.

## 4 Discussion

There are limited pre‐clinical models that examine the efficacy of chemotherapy while also assessing the wellbeing of immunocompetent rodents. This study sought to close the gap by investigating the use of different MTX schedules to balance toxicity and efficacy in a well‐known rat breast cancer model. Our study highlights the challenges associated with optimizing chemotherapy dosing scheduled in the preclinical environment, with narrow therapeutic windows that are also acceptable with respect to toxicity. Despite this, we identified that 2 mg/kg administered every 7 days allows for a longer duration model, providing more time to investigate interventions and observe extended tumor dynamics. Alternatively, the 0.75 mg/kg MTX dose schedule maintains rat welfare while still allowing for efficacy endpoints over a slightly shorter duration.

Compared to our current study, single‐dose chemotherapy models in rats limit the ability to study long‐term effects and cumulative toxicity [4]. Multi‐cycle chemotherapy studies using tumor‐naïve rats are less useful for solid cancer research. Additionally, some models may not adequately address animal welfare concerns, such as the incidence and severity of diarrhea [10]. The MTX dose schedules from our study can be optimized for different experiments, offering several advantages over other models. Our new regimen better mimics human treatment by incorporating multiple doses over time, allowing for more accurate assessment of tumor response and cumulative toxicity. The DAMA model demonstrates tumor growth and regression, aligning with human disease dynamics. It also enables monitoring of side effects relevant to clinical outcomes, while humane endpoints help balance scientific value with animal welfare. Although the model introduces greater burden, its improved translational relevance justifies its use, provided ethical oversight continues. While this report highlights the ability to concurrently model efficacy and toxicity, these findings should be acknowledged in light of some limitations, in particular the small sample size and variation in tumor response to MTX. Timing the first MTX dose relative to tumor size is also challenging, as DAMA tumors grow rapidly; this causes variation in tumor size at the first dose. Future studies should focus on the optimal tumor size (ideally < 0.5%BW) at the first MTX treatment to better understand its influence on model longevity and tumor growth trajectories. Another limitation of this study is the exclusive use of methotrexate, which limits direct comparison with combination regimens but allows clearer interpretation of its standalone efficacy and toxicity.

In conclusion, we have developed a cyclical model of MTX dosing that allows for robust, longitudinal analysis of survival, efficacy, and toxicity concurrently. Tumor control and welfare are both considered moderate, allowing for chemotherapy adjuncts to be tested for their ability to further enhance tumor control or supportive care interventions to be evaluated for their impact on chemo‐efficacy.

## Author Contributions

**Joanne M. Bowen:** conceptualization, investigation, funding acquisition, writing – review and editing, supervision, resources, project administration, methodology. **Hannah R. Wardill:** supervision, project administration, writing – review and editing, validation, conceptualization, funding acquisition, methodology, resources. **Emma Bateman:** conceptualization, methodology, data curation, project administration, investigation, visualization, writing – review and editing. **Ifeoma J. Dikeocha:** conceptualization, investigation, writing – original draft, methodology, visualization, software, formal analysis, data curation, validation, writing – review and editing.

## Funding

This work was supported by Danone Nutricia Research. A/Prof Hannah Wardill is supported by the Hospital Research Foundation Group and NHMRC in the form of a Research Fellowship.

## Conflicts of Interest

The authors declare no conflicts of interest.

## Data Availability

The data that support the findings of this study are available from the corresponding author upon reasonable request.
